# Supplementary material for: Dissecting crosstalk induced by cell-cell communication using single-cell transcriptomic data
Source: Nat Commun. 2025 Jul 1;16:5970. doi: 10.1038/s41467-025-61149-7 (PMC12219092; doi:10.1038/s41467-025-61149-7)
Supplement: Supplementary file 1 — Reporting Summary [file 41467_2025_61149_MOESM1_ESM.pdf]

nature portfolio

Corresponding author(s): Qing Nie

Last updated by author(s): May 18, 2025

Reporting Summary

Nature Portfolio wishes to improve the reproducibility of the work that we publish. This form provides structure for consistency and transparency in reporting. For further information on Nature Portfolio policies, see our Editorial Policies and the Editorial Policy Checklist.

Statistics

For all statistical analyses, confirm that the following items are present in the figure legend, table legend, main text, or Methods section.

n/a	Confirmed	
☐	☒	The exact sample size (*n*) for each experimental group/condition, given as a discrete number and unit of measurement
☐	☒	A statement on whether measurements were taken from distinct samples or whether the same sample was measured repeatedly
☐	☒	The statistical test(s) used AND whether they are one- or two-sided<br>*Only common tests should be described solely by name; describe more complex techniques in the Methods section.*
☒	☐	A description of all covariates tested
☒	☐	A description of any assumptions or corrections, such as tests of normality and adjustment for multiple comparisons
☐	☒	A full description of the statistical parameters including central tendency (e.g. means) or other basic estimates (e.g. regression coefficient) AND variation (e.g. standard deviation) or associated estimates of uncertainty (e.g. confidence intervals)
☐	☒	For null hypothesis testing, the test statistic (e.g. *F*, *t*, *r*) with confidence intervals, effect sizes, degrees of freedom and *P* value noted<br>*Give P values as exact values whenever suitable.*
☒	☐	For Bayesian analysis, information on the choice of priors and Markov chain Monte Carlo settings
☒	☐	For hierarchical and complex designs, identification of the appropriate level for tests and full reporting of outcomes
☐	☒	Estimates of effect sizes (e.g. Cohen's *d*, Pearson's *r*), indicating how they were calculated

Our web collection on statistics for biologists contains articles on many of the points above.

Software and code

Policy information about availability of computer code

Data collection	No software was used for data collection.
Data analysis	SigXTalk is implemented on R and Python. The source code and the tutorial of SigXTalk are publicly available at the following GitHub repository: https://github.com/LithiumHou/SigXTalk and the Zenodo: https://zenodo.org/records/15453916. The code that is used for reproducing the results in the article is publicly available at the following GitHub repository: https://github.com/LithiumHou/SigXTalk_scripts.<br><br>The following programming languages and versions were used:<br>R 4.3.1<br>Python 3.8.12<br><br>The following R software packages were used:<br>Seurat 5.1.0<br>CellChat 2.1.1<br>tidyr 1.3.1<br>dplyr 1.1.4<br>tidymodels 1.2.0<br>Matrix 1.6-5<br>rlang 1.1.4<br>recipes 1.1.0<br>ranger 0..0

parsnip 1.2.1
doParallel 1.0.17
snow 0.4-4
ggplot2 3.5.1
ComplexHeatmap 2.18.0
hrbrthemes 0.8.7
circlize 0.4.16
ggalluvial 0.12.5
ggraph 2.2.1
igraph 2.0.3
ggridges 0.5.6
patchwork 1.2.0
The following Python software packages were used:
pandas 2.0.3
scikit-learn 1.3.0
scipy 1.10.1
numpy 1.24.3
argparse 1.4.0
dhg 0.9.5
torch 1.13.1

For manuscripts utilizing custom algorithms or software that are central to the research but not yet described in published literature, software must be made available to editors and reviewers. We strongly encourage code deposition in a community repository (e.g. GitHub). See the Nature Portfolio guidelines for submitting code & software for further information.

Data

Policy information about availability of data

All manuscripts must include a data availability statement. This statement should provide the following information, where applicable:

- Accession codes, unique identifiers, or web links for publicly available datasets
- A description of any restrictions on data availability
- For clinical datasets or third party data, please ensure that the statement adheres to our policy

All the single-cell RNA-seq datasets and spatial transcriptomic datasets used are publicly available and from previous publications, and no new experimental data is generated in this study. The gut lineage dataset used in this study is available in the Gene Expression Omnibus (GEO, https://www.ncbi.nlm.nih.gov/geo) under accession code GSE152325 [https://www.ncbi.nlm.nih.gov/geo/query/acc.cgi?acc=GSE152325]. The HNSCC dataset used in this study is available in GEO under accession code GSE103322 [https://www.ncbi.nlm.nih.gov/geo/query/acc.cgi?acc=GSE103322], or at Zenodo (https://zenodo.org/records/3260758). The COVID-19 dataset used in this study is available via the single-cell portal (https://singlecell.broadinstitute.org/single_cell/study/SCP1219); note that sign-in is required to access the data. The mouse lung dataset used in this study is available in GEO under accession code GSE141259 [https://www.ncbi.nlm.nih.gov/geo/query/acc.cgi?acc=GSE141259]. The mouse brain spatial transcriptomics dataset can be accessed at the 10x genomics (https://www.10xgenomics.com/resources/datasets/mouse-brain-serial-section-1-sagittal-anterior-1-standard-1-0-0).

Research involving human participants, their data, or biological material

Policy information about studies with human participants or human data. See also policy information about sex, gender (identity/presentation), and sexual orientation and race, ethnicity and racism.

Reporting on sex and gender	N/A. We used public data in the manuscript and there is no human research participants involved.
Reporting on race, ethnicity, or other socially relevant groupings	N/A. We used public data in the manuscript and there is no human research participants involved.
Population characteristics	N/A. We used public data in the manuscript and there is no human research participants involved.
Recruitment	N/A. We used public data in the manuscript and there is no human research participants involved.
Ethics oversight	N/A. We used public data in the manuscript and there is no human research participants involved.

Note that full information on the approval of the study protocol must also be provided in the manuscript.

Field-specific reporting

Please select the one below that is the best fit for your research. If you are not sure, read the appropriate sections before making your selection.

[x] Life sciences [ ] Behavioural & social sciences [ ] Ecological, evolutionary & environmental sciences

For a reference copy of the document with all sections, see nature.com/documents/nr-reporting-summary-flat.pdf

Life sciences study design

All studies must disclose on these points even when the disclosure is negative.

Sample size	No statistical sample size calculation was performed for this study. The sample sizes of scRNA and/or spatial transcriptomics data were determined based on the tissue slides in practical measurements.
Data exclusions	Quality control was performed for each dataset used in this study. Cells with abnormally high RNA counts or mitoRNA counts were removed.
Replication	All attempts at replication were successful and included in data analyses.
Randomization	Randomized sampling were used in the benchmark using simulated data. At least 10 independent repeated tests were performed for a single experiment to ensure that the randomization would not significantly affect the results.
Blinding	N/A

Reporting for specific materials, systems and methods

We require information from authors about some types of materials, experimental systems and methods used in many studies. Here, indicate whether each material, system or method listed is relevant to your study. If you are not sure if a list item applies to your research, read the appropriate section before selecting a response.

Materials & experimental systems

n/a	Involved in the study
☒	☐ Antibodies
☒	☐ Eukaryotic cell lines
☒	☐ Palaeontology and archaeology
☒	☐ Animals and other organisms
☒	☐ Clinical data
☒	☐ Dual use research of concern
☒	☐ Plants

Methods

n/a	Involved in the study
☒	☐ ChIP-seq
☒	☐ Flow cytometry
☒	☐ MRI-based neuroimaging

Plants

Seed stocks	N/A
Novel plant genotypes	N/A
Authentication	N/A
